# Mechanical and Thermal Properties of Wood-Fiber-Based All-Cellulose Composites and Cellulose-Polypropylene Biocomposites

**DOI:** 10.3390/polym15030475

**Published:** 2023-01-17

**Authors:** Eija-Katriina Uusi-Tarkka, Mikael Skrifvars, Pooria Khalili, Henrik Heräjärvi, Nawar Kadi, Antti Haapala

**Affiliations:** 1 School of Forest Sciences, Faculty of Science and Forestry, University of Eastern Finland, FI-80101 Joensuu, Finland; 2 Swedish Centre for Resource Recovery, Faculty of Textiles, Engineering and Business, University of Borås, SE-50190 Borås, Sweden; 3 Natural Resources Institute Finland, FI-80100 Joensuu, Finland; 4 Department of Textile Technology, Faculty of Textiles, Engineering and Business, University of Borås, SE-50190 Borås, Sweden; 5 FSCN Research Centre, Mid Sweden University, SE-85170 Sundsvall, Sweden

**Keywords:** ACC, laminate, mechanical performance, NaOH–urea solvent, single-polymer composite, sustainability, textile structures, thermal analysis, wood fibers

## Abstract

This article explores wood-fiber-based fabrics containing Lyocell yarn in the warp and Spinnova–Lyocell (60%/40%) yarn in the weft, which are used to form unidirectional all-cellulose composites (ACC) through partial dilution in a NaOH–urea solution. The aim is to investigate the role of the yarn orientation in composites, which was conducted by measuring the tensile properties in both the 0° and 90° directions. As a reference, thermoplastic biocomposites were prepared from the same fabrics, with biobased polypropylene (PP) as the matrix. We also compared the mechanical and thermal properties of the ACC and PP biocomposites. The following experiments were carried out: tensile test, TGA, DSC, DMA, water absorption test and SEM. The study found no significant difference in tensile strength regarding the Spinnova–Lyocell orientation between ACC and PP biocomposites, while the composite tensile strength was clearly higher in the warp (Lyocell) direction for both composite variants. Elongation at break doubled in ACC in the Lyocell direction compared with the other samples. Thermal analysis showed that mass reduction started at a lower temperature for ACC, but the thermal stability was higher compared with the PP biocomposites. Maximum thermal degradation temperature was measured as being 352 °C for ACC and 466 °C for neat PP, and the PP biocomposites had two peaks in the same temperature range (340–474 °C) as ACC and neat PP combined. ACCs absorbed 93% of their own dry weight in water in just one hour, whereas the PP biocomposites BC2 and BC4 absorbed only 10% and 6%, respectively. The study highlights the different properties of ACC and PP reference biocomposites that could lead to further development and research of commercial applications for ACC.

## 1. Introduction

Environmental concerns, such as dependence on petroleum-based materials, increasing amounts of nonbiodegradable waste and pollution, are among the growing concerns that have driven new biomaterials development [1]. Substituting petroleum-based polymers with natural fibers or other organic sources has resulted in an increasing interest in producing various composites from renewable, nonfossil resources. Cellulose-reinforced composites can be an environmentally friendly option because they originate from renewable resources and have good biodegradability, have low density, are cost effective, have good mechanical performance and are abundant [2,3].

As a semicrystalline polymer, cellulose is difficult to dissolve [4]. During heating, its crystalline phase is stabilized by strong hydrogen bonds, so the cellulose does not go through crystal melting [5]. Solvents that can disturb the intramolecular bonding of cellulose can offer a route to process it into useful products, such as regenerated fibers. Currently, two industrially dominant technologies for creating regenerated cellulose fibers are the Viscose and Lyocell processes [5]. Regenerated fibers are characterized by their high purity, uniformity and reproducibility of mechanical and thermal properties [6]. There are also numerous new types of wood fibers under development and in the early stages of commercial production for the textile industry, where the focus is on improving production efficiency, waste management and recycling, reducing the use of chemicals and water and creating closed-loop production [7,8]. One new generation wood fiber is the Spinnova fiber, which is mechanically refined without dissolution or regeneration from microfibrillated cellulose that originates from raw wood pulp. Spinnova fiber is often combined with other fibers, such as Lyocell, when it is woven into a textile material. Spinnova–Lyocell textile reinforcements could be used in composite manufacturing in the same way as other cellulosic reinforcements, but because it is a new fiber product, limited academic research has yet been conducted.

The interaction between the reinforcement and matrix is crucial for the mechanical and thermal properties of the composites. Thermoplastic polymers, such as polypropylene, are not fully compatible with highly hydrophilic cellulose because of cellulose’s abundant hydroxyl groups [9]. The weak interface between the reinforcement and matrix can be improved by physical methods that change the structural and surface properties of the fiber (e.g., surface fibrillation and electric discharge) or by chemical methods (e.g., pretreatments, coupling agents and graft copolymerization) [10,11]. However, treatments usually add costs and can increase chemical intake, as well as creating harmful pollution [12]. An alternative solution to enhance adhesion between the components is to create a single-polymer composite, in which both the reinforcement and matrix are formed by using the same polymer [13,14].

All-cellulose composites (ACCs) are single-polymer composites formed of any type of cellulose. Biodegradable ACCs can be produced by using two different cellulosic sources, which are responsible for forming both the reinforcement and matrix. Another option is to partly dissolve cellulose fibers so that the dissolved cellulose will form a matrix around the reinforcement when the composite is processed by compression and heating. Diverse selections of ACCs can be produced using different methods, solvents and raw materials [8,15]. Nevertheless, less research has been published so far on comparing properties of textile-based materials fabricated either as ACCs or as traditional biocomposites. Finding out how the properties of ACCs stand compared with biocomposites is highly important for further development of the ACCs and for their possibilities in the application range.

The aim and novelty of the present study was to investigate the performance of Lyocell and less-known Spinnova fibers in ACC materials compared with polypropylene matrix composites and to identify the advantages and disadvantages that ACC materials can yield. Because it is known that the direction of the fiber vastly contributes to the properties of the composite [16], unidirectional laminates were examined with a tensile tester for two directions (0° and 90°) to receive a direct comparison of the tensile behavior in the ACC and biocomposites. Thermal analysis and water absorption tests were implemented to gain further knowledge of the composite’s performance under changing conditions, which can also greatly affect the material’s demand.

## 2. Materials and Methods

### 2.1. Materials and Treatments

**Figure 1:**
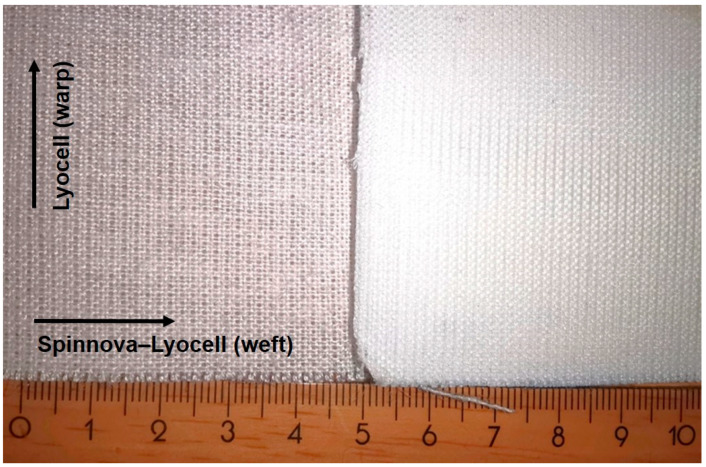
Initial material: Spinnova-Lyocell is seen on the left and produced ACC on the right.

The textile fabric used in the present study was a plain weave with two-ply Lyocell yarn with 12.6 ends/cm in the warp and 60/40% Spinnova–Lyocell yarn with 16.5 picks/cm in the weft with a surface weight of 173 g/m^2^. The material was provided by Spinnova Ltd., (Jyväskylä, Finland). The structure of the used textile fabric and the corresponding obtained ACC composite is shown in Figure 1.

An aqueous NaOH–urea solvent was used to partially dissolve the cellulosic fiber structures. The solvent was prepared by dissolving 12 wt.-% NaOH and 7 wt.-% urea in water at room temperature. The solution was then cooled down to −12 °C and used to prepare the composites by soaking the assembled fabrics in a volume of 0.6 liters. The same solvent batch was used to produce three to four laminate samples. The chemicals were of reagent grade and were supplied by Sigma-Aldrich (Darmstadt, Germany). A biobased (up to 30%) polypropylene was supplied by Nature Plast (Caen, France) and was used to produce the reference PP biocomposites.

### 2.2. Composite Laminate Preparation

The all-cellulose composite laminates were produced by layering four sheets of fabrics (18 × 18 cm^2^) unidirectionally as a stack. The unidirectional yarn orientation was controlled by a mark on the individual layers. The piles were fully impregnated with the aqueous NaOH–urea solvent for 1 min. Each laminate pile rapidly absorbed around 150 g of the solvent, which was determined by weighing the fabric pile before and after impregnation. The impregnated fabric piles were then placed in 10 L buckets and carefully rinsed with water to remove excess solvent. After rinsing, the samples were left in the buckets in fresh water. Water was changed repeatedly until the pH of the surface of the samples became neutral, as tested using indicator paper. Neutralization took approximately 48 h. If the stacked layers disintegrated during the dissolution or rinsing process, they were manually joined together by tweezers before further processing.

The partly dissolved fabrics were converted to solid composites by pressing the fabric layers together in a hydraulic hot press, where the temperature was set to 100 °C and pressure to 60 bar. The pressing cycles are presented in Table 1. The samples were first pressed for 5 s four times to remove excess water. This was followed by applying 1 min pressure three times, with a 10 s steam release period between the pressings. To continue processing, the samples were pressed once for 3 min and twice for 5 min, here with a 20 s steam release period between the pressings. Finally, the samples were placed in the press for 20 min at 100 °C. After the hot press procedure, the ACC composites (average thickness of 0.60 mm) were wrapped in tissue paper and left between metal plates (fixed hand-tight with screws) overnight at room temperature.

Thermoplastic biocomposite reference laminates were manufactured by combining the same Spinnova–Lyocell textile with polypropylene (PP). PP granulates were transformed to PP films with the hot press (200 °C), giving films with an approximate thickness of 0.25–0.40 mm. Thinner biocomposites (with average thickness of 0.68 mm) had two layers of cellulose fabric and two layers of PP films (denoted as BC2), and thicker biocomposites (with average thickness of 1.29 mm) had four layers of cellulose fabric and four layers of PP film (denoted as BC4). Each laminate had approximately 45 wt.-% of cellulosic textile fabric and 55 wt.-% of PP. The biocomposites were composed of alternating layers of cellulose fabrics and PP films. The compression molding was carried out in a hot press at 200 °C for 2 min. The obtained biocomposite laminates were finally placed at room temperature between the metal plates for at least 15 min to allow for complete solidification of the PP matrix.

### 2.3. Composite Characterisation

#### 2.3.1. Tensile Test

Tensile tests were carried out in accordance with the ISO 527 standard using a Tinius Olsen H10KT (Horsham, PE, USA) testing machine. A mechanical extensometer attached to the specimen was used to measure the strain. The rate of loading was 10 mm/min and the load tension was 2.5 kN. The gauge length was 50 mm, and the initial distance between grips was 115 mm. The dumbbell-shaped specimens were cut from the laminates using a laser cutting machine Laser Pro, GLS (Taipei, Taiwan). Tensile tests were performed in two directions (0° and 90°) to determine how fiber orientation affects the mechanical properties of the composites. Twelve replicate specimens were tested in both yarn directions and for all laminate types.

#### 2.3.2. Thermogravimetric Analysis

Thermogravimetric analysis (TGA) and differential thermogravimetric analysis (DTGA) were conducted by an SDT Q500 thermal analyzer (TA instrument, New Castle, DE, USA). Samples of approximately 12 mg were heated at 10 °C/min from 30 °C to 650 °C in a nitrogen environment. The nitrogen flow rate was set to 40 mL/min.

#### 2.3.3. Differential Scanning Calorimetry

Differential scanning calorimetry (DSC) was carried out using the DSC Q1000 instrument (TA instruments, New Castle, DE, USA). An approximately 7 mg sample was heated in a nitrogen purge steam at a heating rate of 10 °C/min from 0 °C to 200 °C, which was followed by cooling and a new heating cycle to determine the glass transition temperature.

#### 2.3.4. Dynamic Mechanical Analysis

Dynamic mechanical analysis (DMA) was performed using a single cantilever test with a Rheometrics Solids Analyzer RSA II (TA Instruments, New Castle, DE, USA). The temperature range was set from 25 °C to 220 °C (note 160 °C for PP). The heating rate was set to 3 °C/min, and the testing frequency was 1 Hz. Test samples were cut to a rectangle shape (50 mm × 10 mm).

#### 2.3.5. Water Absorption Test

A water absorption test was carried out on samples of size 10 × 80 mm^2^. The samples were first dried in an oven for 24 h at 60 °C, and then their mass was measured (denoted as *W*_0_). During the first day, two additional measurements were taken: weight after 1 h and 3 h of water exposure. The next measurements were scheduled after 24 h for the next 6 days. The mass at each measurement point in time was compared with *W*_0_ to detect the amount of water absorbed as a function of time. The mass gain (*m*) is the percentage change in mass relative to the initial mass, which was calculated using Equation (1). The average mass gain was calculated from five replicated samples.

(1) $$m=\frac{W-W_{0}}{W_{0}}\cdot 100\%$$

#### 2.3.6. Scanning Electron Microscopy

Morphological changes in the cross-section area were detected using scanning electron microscope (SEM) imaging carried out using a Hitachi S-4800 (Hitachi, Tokyo, Japan). The samples were gold-coated (2 nm) with a Cressington sputter coater 208 HR (Watford, UK) before the imaging.

## 3. Results and Discussion

### 3.1. Tensile Test

Tensile tests were carried out in two directions, where S stands for the Spinnova–Lyocell (weft) direction and L stands for the Lyocell (warp) direction. The thinner polypropylene reference biocomposites were marked as S-BC2 and L-BC2, and the thicker polypropylene biocomposites were marked as S-BC4 and L-BC4. ACCs were marked as S-ACC and L-ACC, depending on the direction of the test.

**Figure 2:**
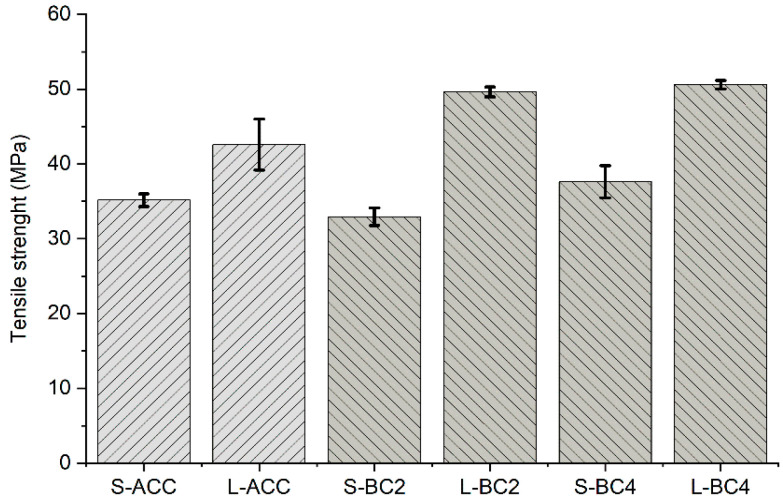
Tensile strength (MPa) for ACC and PP reference biocomposites. Tensile strength measurements were performed in two directions, where S stands for the Spinnova–Lyocell (weft) direction and L stands for the Lyocell (warp) direction. Lighter shade is used for ACC, darker for BC.

The tensile strength (MPa) results show (Figure 2) that there was practically no difference between the ACC and PP biocomposites, BC2 and BC4, measured in the Spinnova–Lyocell direction; indeed, the ACCs and biocomposites had quite similar strengths (35 +/− 2 MPa). All the composites had higher tensile strength in the Lyocell direction compared with the Spinnova–Lyocell direction, indicating better mechanical strength for the Lyocell fibers. This could be explained by the Spinnova fiber being more dissolved in the NaOH–urea dissolution process than the Lyocell fiber. The composite will therefore have better strength in the Lyocell direction. The highest tensile strength, 51 MPa, was measured for the L-BC4, and the lowest, 43 MPa, for the ACC in the Lyocell direction.

A study comparing ACCs and biocomposites was published by Gildl-Altmutter et al. [17], who reported a tensile strength of 34 MPa for nonwoven flax-based ACC, which is comparable to the 35 MPa value for S-ACC seen in our experiment. However, in their study, the tensile strength for flax–epoxy biocomposites was measured as being 79 MPa, which is more than double compared with their flax-based ACC. In our study, the difference between ACC and reference PP biocomposites in the Lyocell direction was only 2 MPa for the Spinnova–Lyocell direction and 7–8 MPa for the Lyocell direction. Nevertheless, comparing our results to other studies is complicated because of differences in the amounts of fibers, their purities, solvents used and manufacturing techniques and conditions.

**Figure 3:**
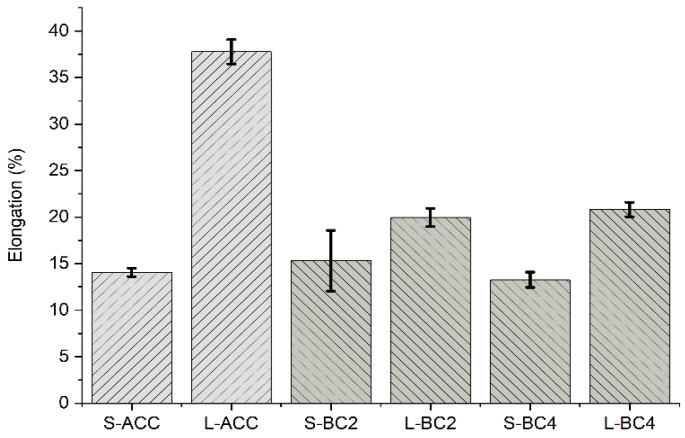
Elongation at break (%) for ACC and biocomposites. Measurements were performed in two directions, where S stands for the Spinnova–Lyocell (weft) direction and L stands for the Lyocell (warp) direction. Lighter shade is used for ACC, darker for BC.

**Figure 4:**
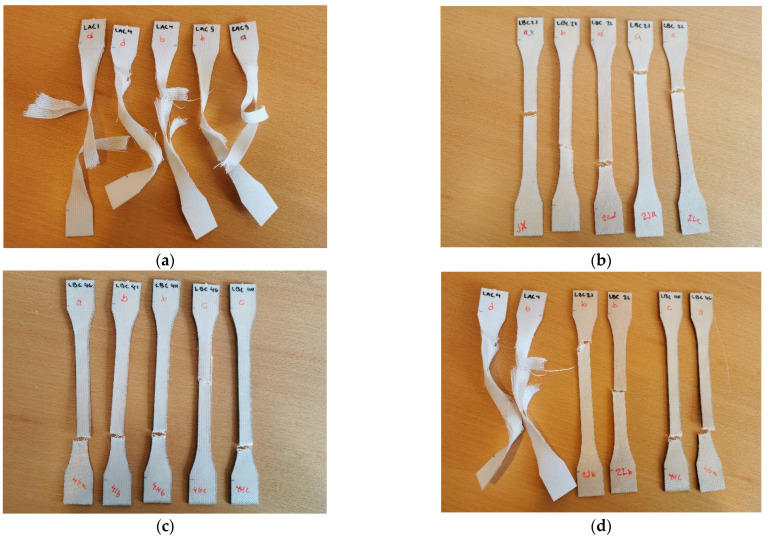
Broken dumbbell tensile test samples. Test samples of L-ACC are seen in (**a**), L-BC2 in (**b**) and L-BC4 in (**c**), and two of each sample category in the same order are seen in image (**d**).

Similarly, the elongation at break (%) did not vary much between the S-ACC, S-BC2 and S-BC4 (Figure 3) specimens. The results also indicate that elongation at break (%) was higher for all composites in the Lyocell direction than in the Spinnova–Lyocell direction. The number of fabric layers, either two or four, in the PP biocomposites did not greatly affect the properties. One interesting result was that elongation at break (%) value for L-ACC was as high as 38%, whereas other composites’ received values were between 13% and 21%. Adak and Mukhopadhyay [18] measured the tensile properties of Lyocell-fabric-based ACC using eight textile layers and ionic liquids as solvents. For a half an hour dissolution time, they received elongation at break values of roughly 26% in the warp direction and 30% in the weft direction. The elongation at break values in our L-ACC specimens were higher than those in the specimens of Adak and Mukhopadhyay [18]. A reason for this might be that Lyocell fiber, which has a skin–core structure [19], is more elastic than the Spinnova fiber, which is produced without regeneration [20]. In addition, L-ACC does not have a cohesive matrix, such as polypropylene in L-BC2 and L-BC4, which will keep the fiber in place and prevent greater elongation. Different textile layers are also strongly attached to each other in the PP biocomposites compared with ACC. This is visualized in the broken tensile test samples (Figure 4).

### 3.2. Thermogravimetric Analysis

Thermogravimetric analysis (TGA) quantified the thermal resistance by recording the percentage weight loss of the samples when there were increasing temperatures. In the thermal analyses, there was no difference between the Lyocell and Spinnova–Lyocell yarn directions; therefore, only each type of composite, that is, ACC, BC2 and BC4, were examined. In addition, neat PP film was tested to give a reference value for the PP biocomposites.

**Figure 5:**
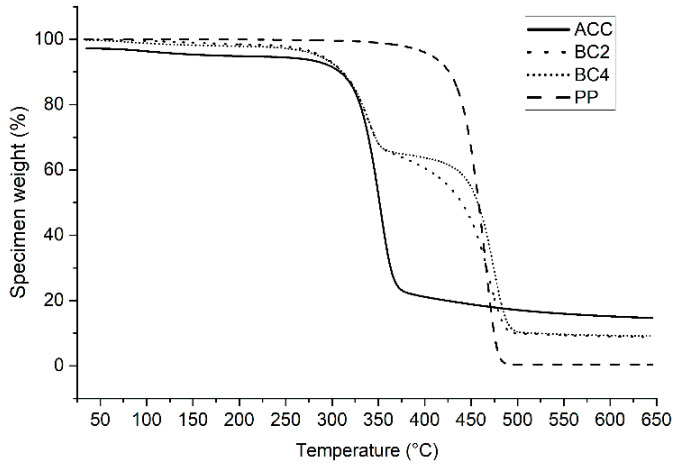
Results of TGA for all-cellulose composites (ACC), polypropylene biocomposites (BC2 and BC4) and neat polypropylene (PP).

The TGA results (Figure 5) show that there was a small mass reduction for ACC during the beginning of the heating cycle, which was because of evaporating water. This was also reported in previous studies [21,22,23]. A further sharp decrease in mass reduction for ACC started at around 310 °C, and a steadier decrease continued at around 360 °C. For PP biocomposites, a sharp mass reduction started later at around 420 °C. It also approached zero in a single step without leaving any significant residue. The PP biocomposites behaved almost identically in TGA, and we could see a clear difference between them and the ACC and neat PP. It was also visible that degradation decreased in the two different temperature regions where the curves were corresponding values of ACC and PP. The biocomposites’ final mass residue was around 10% in the end; therefore, compared with PP, it had higher thermal stability, while ACC had the highest.

**Figure 6:**
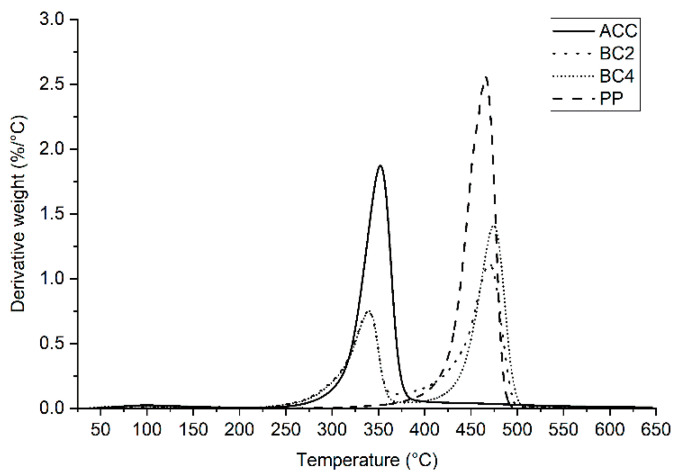
Results of the DTGA for all-cellulose composites (ACC), biocomposites (BC2 and BC4) and polypropylene (PP).

Figure 6 shows the results of the differential thermogravimetric analysis (DTGA). The maximum thermal degradation temperature was measured at 352 °C for ACC and 466 °C for PP biocomposites. For the biocomposites, thermal degradation occurred in a similar temperature range (from 340 °C to 474 °C) than ACC and PP. The first peaks indicating maximum thermal degradation were attributed to the cellulose reinforcement, and the second peaks were attributed to the PP polymer matrix. The maximum decomposition rates were close to the ACC and PP values, but there was a small shift where the high-intensity areas were located, which could be because of the changes in mass. The values of the first peaks for BC2 and BC4 were 339 °C and 340 °C and, for the second peaks, 469 °C and 475 °C, respectively.

Measured values from thermal testing depend on parameters such as nitrogen rate, sample weight and heating rate [24]. In a nitrogen environment, the thermal decomposition of PP has previously been recorded to start at 300 °C and end at 475 °C [25], which is ~10 °C lower than what was recorded in our study. Yang et al. [22] manufactured ACCs from regenerated fibers and reported that the initial decomposition temperatures of modified ramie fibers, natural ramie fibers and regenerated cellulose films were 281 °C, 262 °C and 270 °C, respectively, while the peak of the modified ramie fibers was approximately 356 °C. The results indicate that ACCs made of Spinnova–Lyocell cellulose fibers in our research have as high thermostability as modified ramie-fiber-based ACCs.

### 3.3. Differential Scanning Calorimetry

**Figure 7:**
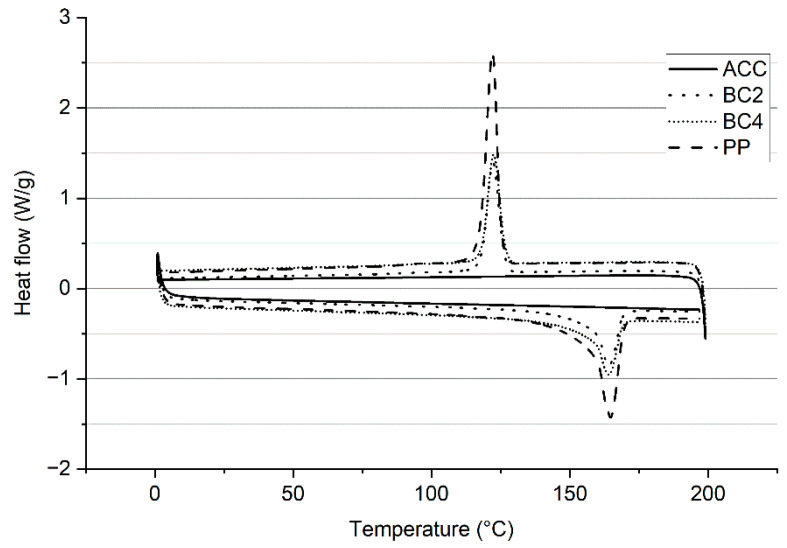
Results of DSC in a range between 0 °C and 200 °C for all-cellulose composites (ACC), biocomposites (BC2 and BC4) and polypropylene (PP).

Differential scanning calorimetry (DSC) can detect any residual exothermal heat present in the samples. As shown in Figure 7, the exothermic peak, which represents crystallization temperature (T_c_), was measured at 122 °C for PP, BC4 and BC2, while the respective heat flows were 2.6 W/g, 1.5 W/g and 1.4 W/g. The melting temperatu©T_m_ for the PP biocomposites and neat PP was measured at 164 °C. Similar results have been reported in the literature: T_m_ was detected at 165 °C for pure PP (Ibrahim et al., 2016) and 163 °C for biocomposites modeled using microcrystalline cellulose and PP/PLA [26]. The main difference between PP, PP biocomposites and ACC was that ACC did not have an explicit T_c_ or T_m_, as expected. Cellulose has a strong network of hydrogen bonds and undergoes thermal decomposition when heated [27,28].

### 3.4. Dynamic Mechanical Analysis

**Figure 8:**
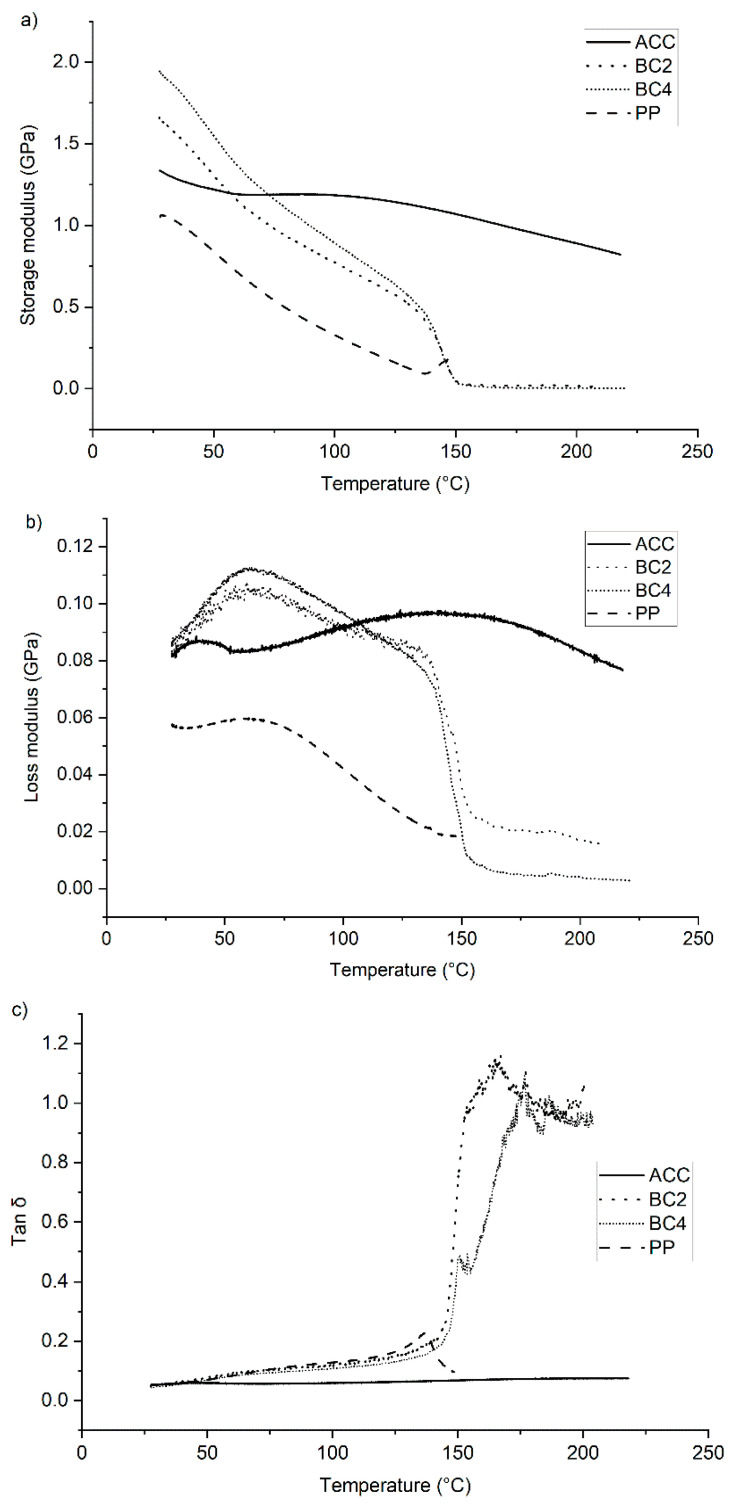
Results of the DMA: (**a**) storage modulus (GPa), (**b**) loss modulus (GPa) and (**c**) tan δ for all-cellulose composites (ACC), biocomposites (BC2 and BC4) and polypropylene (PP).

The dynamical mechanical analysis (DMA) measures the viscoelastic properties of the samples. The results are divided into three categories: storage modulus (GPa) (Figure 8a), loss modulus (GPa) (Figure 8b) and tan delta (δ) (Figure 8c). According to DSC, the melting temperature for PP was 164 °C. Therefore, the analysis was programmed to end at 150 °C for PP, while it lasted longer (~220 °C) for the other samples.

The storage modulus represents the energy stored in the elastic structure of the sample (Frank, TA instruments). During the beginning of the test (25 °C), the thickest PP biocomposite with four layers of fabric (BC4) had the highest storage modulus (1.9 GPa), followed by BC2 (1.7 GPa), ACC (1.3 GPa) and pP (1 GPa). The PP and reference PP biocomposites had a steady decrease in storage modulus until 150 °C. When these samples approached the melting temperature of PP, the storage modulus remained at zero. ACC lost less than 40% of its storage modulus during the experiment (from 1.3 GPa to 0.8 GPa). This phenomenon, where ACCs’ storage modulus remained high and relatively steady, has also been reported previously by Soykeabkaew and Peijis [29]. According to the results, the viscoelastic properties of PP biocomposites BC2 and BC4 did not resemble the properties of ACC but rather the properties of neat PP, which was expected. The structure of the biocomposites and their ability to elastically respond to stress was lost when PP reached near T_m_.

The loss modulus represents the viscous part, or the amount of energy dissipated in the sample [30]. Evaporating water in ACC created a small reduction in the loss modulus, but overall, the values stayed high when compared with neat PP and PP biocomposites. Thermal softening and a decrease in loss modulus started to gradually occur for the neat PP and PP biocomposites at 60 °C, which was followed by a sharp decrease at 140 °C for the PP biocomposites. BC4 had a higher amount of cellulose fabric but also a higher amount of PP, which can be seen as a lower loss modulus after 150 °C compared with BC2. The behavior of the PP matrix influenced the thermal properties of biocomposites more strongly than its cellulosic filler.

Tan delta is the ratio between the loss modulus and storage modulus. The PP and PP biocomposite results are not meaningful to compare after PP has reached its limits regarding viscoelastic stress, since once this deformation of the composites has taken place, the material will not recover to its original form. The glass transition temperature (T_g_) for polypropylene varies depending on its crystallinity, but it is recorded to be below −25 °C [31], around 0 °C [32] or just above −12 °C [33]. In this graph, T_g_ is not detectable for neat PP, PP biocomposites, or ACC. Even though cellulose underwent a second-order phase transition from the low-temperature glass state to the high-temperature elastic state [34,35], earlier research has reported that there was not necessarily a clear T_g_ for cellulose [17,36], as was the case here.

### 3.5. Water Absorption Test

**Figure 9:**
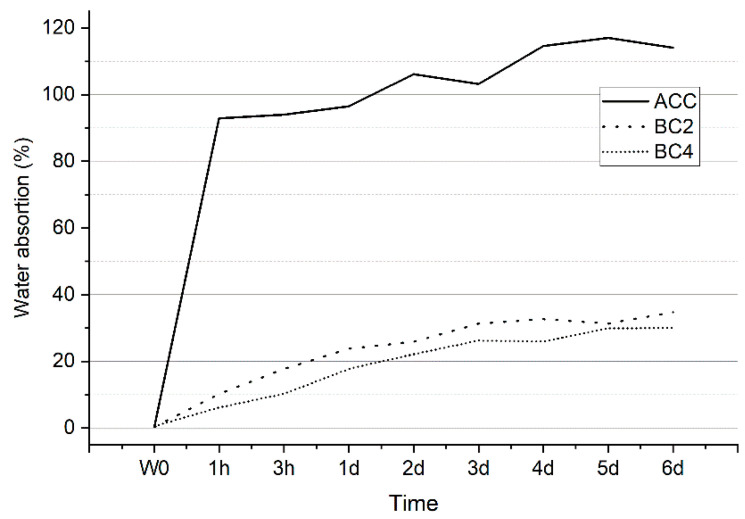
Results from a water absorption test of 6 days, where W_0_ stands for dry weight.

Figure 9 shows the weight gain (%) of the composites as a function of water immersion time. The graph shows that all composites absorbed some water after immersion. However, there was a major difference in the water absorption rate between ACC and PP biocomposites already after 1 h. During this first hour, ACC absorbed 93% of its own dry weight in water, whereas BC2 and BC4 absorbed only 10% and 6%, respectively. After a day, ACC reached 97%, meaning that composites almost doubled their weight in 24 h. On the fourth day, all the composites had absorbed close to their maximum capacity of water, and the absorption rate leveled off during the following days. The maximum water absorption values were reached on day 5 for ACC (117%) and BC4 (30%) and on day 6 for the BC2 (35%). There was no considerable difference between BC2 and BC4, but water intake was slightly lower for BC4. The share of hydrophobic PP matrix was higher in BC4, which likely contributed to the composites’ low water absorption rate. Hydrophilic ACCs’ tendency to absorb water will greatly limit their possible applications without a waterproof coating or modification.

### 3.6. Scanning Electron Microscopy

**Figure 10:**
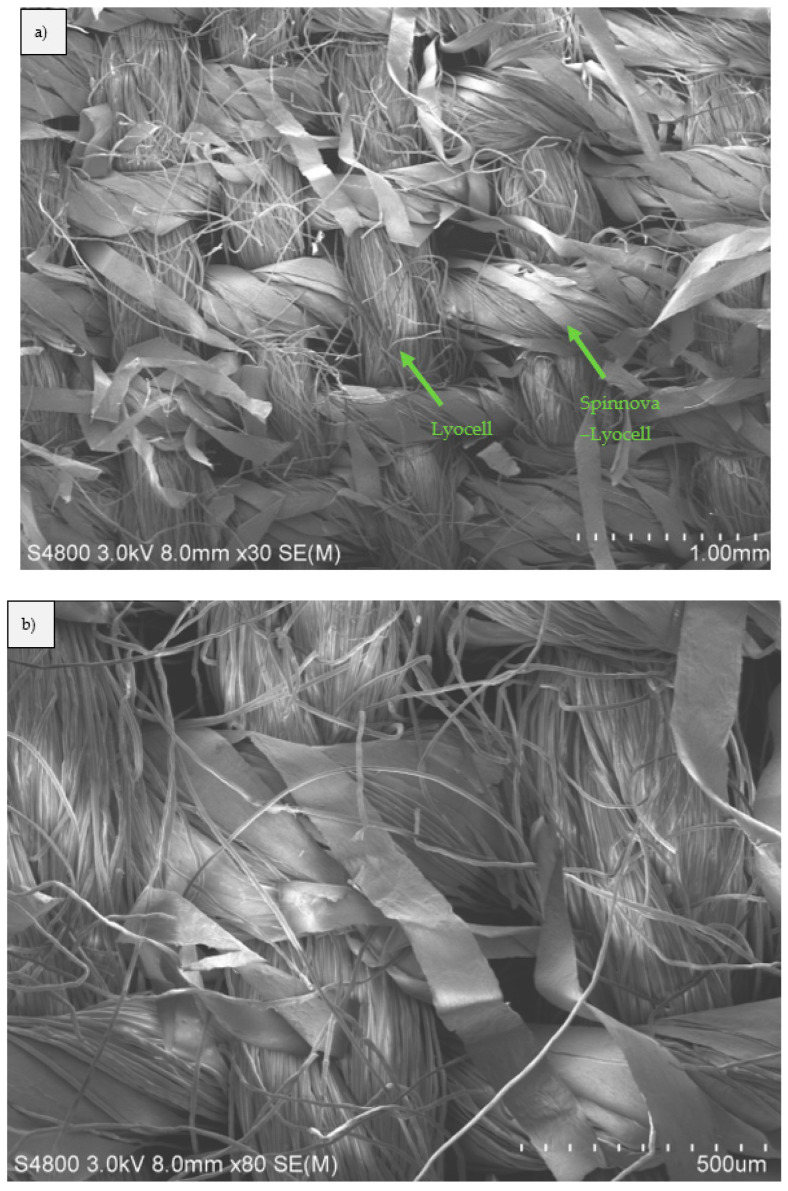
SEM pictures of Spinnova–Lyocell fabric with Lyocell yarn in the warp (longitudinal direction) and 60/40% Spinnova–Lyocell yarn in the weft (horizontal direction), with magnifications of (**a**) 30× and (**b**) 80×.

**Figure 11:**
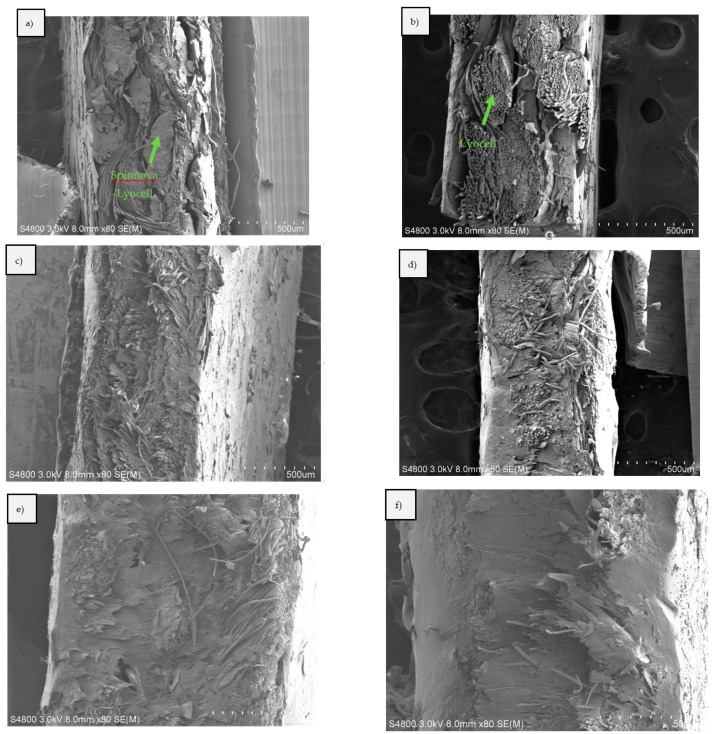
SEM pictures of the composites: (**a**) S-ACC, (**b**) L-ACC, (**c**) S-BC2, (**d**) L-BC2, (**e**) S-BC4 and (**f**) L-BC4, with a magnification of 80×.

**Figure 12:**
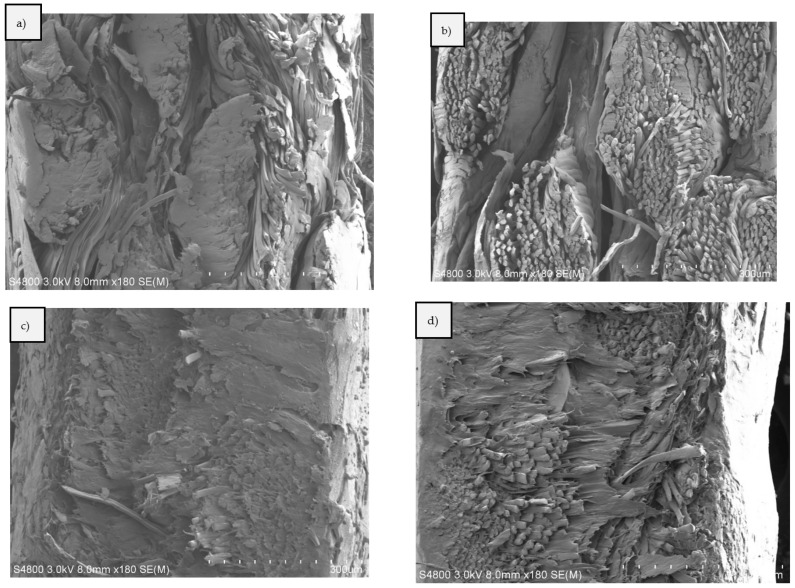
SEM pictures of the composites: (**a**) S-ACC, (**b**) L-ACC, (**c**) S-BC2 and (**d**) L-BC2, with a magnification of 180×.

Scanning electron microscopy (SEM) pictures were taken from the neat Spinnova–Lyocell fabric (Figure 10). The different fiber structures and width of the yarns are visible in the images. Cross-sectional SEM pictures of S-ACC, L-ACC, S-BC2, L-BC2, S-BC4 and L-BC4 composites can be seen in Figure 11, here with a magnification of 80×. Figure 12 presents the S-ACC, L-ACC, S-BC2 and L-BC2 structures with a magnification of 180×. All images to the left (Figure 11a,c,e and Figure 12a,c) are composite cross-sections with the Spinnova–Lyocell fibers in the horizontal direction (out of plane) and the Lyocell fibers in the vertical direction. All images on the right side (Figure 11b,d,f and Figure 12a,c) have the fibers in opposite direction. A clear difference can be seen between images (a) S-ACC and (b) L-ACC in Figure 10 and Figure 11. During composite processing, the Spinnova fiber dissolved in greater extent compared with the Lyocell fiber. In the image of S-ACC, (a) the Spinnova fibers are well fused together, but in L-ACC (b) individual undissolved Lyocell fibers are still visible as independent fibers. In both ACC images, different layers of textile fabrics are clearly visible. In the case of the reference PP biocomposites (In Figure 11c–f and Figure 12c,d), melted PP has shaped the structure of the composite so that cellulosic fibers or individual layers of the textile cannot be clearly detected anymore. This is even more evident in the thicker composites S-BC4 (Figure 11e) and L-BC4 (Figure 12f).

## 4. Conclusions

The current study revealed that the direction of the studied yarns greatly contributed to the composites’ tensile strength and elongation properties. There was no considerable difference in the tensile strength between the ACC and PP reference biocomposites when measured in the Spinnova–Lyocell yarn direction, whereas when measured in the Lyocell yarn direction, the tensile strength was higher in the PP reference biocomposites than in the ACCs. Overall, the Lyocell-reinforced composites had better tensile strength compared with the Spinnova–Lyocell-reinforced composites. The main finding in elongation studies was that the L-ACC composite has a remarkably higher elongation at break (38%) compared with any of the other composites. Regenerated Lyocell seems to have very textile-like characteristics, even as ACC, whereas in the reference biocomposites, the stiffness of the PP matrix prevented greater elongation. In addition, the individual reinforcement layers in the ACC were not as well bonded to each other as they were in the reference PP biocomposites. A challenge in the development of ACCs is therefore to prevent the unwanted delamination of the textile layers. With PP biocomposites, the orientation of the fibers had more impact on the properties of the composite than the number of polymer layers used.

The thermal studies demonstrated that ACCs had a better thermal stability than the PP biocomposites. PP dominated the thermal behavior in the reference biocomposites, which was expected. T_c_ and T_m_ values of 122 °C and 164 °C, respectively, were detected for the PP biocomposites, whereas no crystallization or melting temperatures were identified for cellulose composites. The high cross-linking of cellulose restricted the movements of the chain segments during heating, which would be required for fluid transformation. Instead, the cellulose degrades at high temperatures and does not show any thermal transitions. The water absorption test showed the highly hydrophilic nature of cellulose: ACC absorbed twice its weight in water in the immersion test in less than 48 h. Because of the protective layer of PP, the biocomposites resisted wetting much better, and there was only a minor difference between BC2 and BC4 composites.

The SEM images showed clear differences in structure for the two studied cellulose fibers. Lyocell and Spinnova–Lyocell had different fiber structures in neat fabrics, which were also detectable in the composites. In the reference biocomposites, especially in the thicker laminates, the different fabric layers could not be as easily seen in the cross-sectional images as in the ACCs. The reported findings regarding the role of direction of the fiber and properties of ACC compared with PP biocomposites are important for further optimization, product development and use in structural composite applications.

## Figures and Tables

**Table 1:** Pressing cycles in composite manufacturing for the hot press process.

Cycle Order	Pressure Time	Release Time	Number of Cycles	Pressure	Temperature
1	5 s	10 s	4	60 bar	100 °C
2	1 min	10 s	3	60 bar	100 °C
3	3 min	20 s	1	60 bar	100 °C
4	5 min	20 s	2	60 bar	100 °C
5	20 min	20 s	1	60 bar	100 °C

## Data Availability

The data presented in this study are available on request from the corresponding author.
